# Pathological diversity of pulmonary sarcoidosis

**DOI:** 10.3389/fmed.2025.1666973

**Published:** 2025-11-27

**Authors:** Tamiko Takemura

**Affiliations:** Department of Pathology, Kanagawa Cardiovascular and Respiratory Center, Yokohama, Kanagawa, Japan

**Keywords:** sarcoidosis, lung, epithelioid cell granuloma, fibrosis, granulomatous angiitis, pathology

## Abstract

Sarcoidosis is a multisystem disease characterized histologically by the presence of non-caseating epithelioid cell granulomas. Although the etiology of sarcoidosis remains uncertain, *Cutibacterium acnes* (formerly known as *Propionibacterium acnes*) and mycobacteria are considered putative pathogenic antigens in sarcoidosis. Sarcoidosis must be differentiated from a variety of granulomatous diseases of the lung. The granulomas in pulmonary sarcoidosis are frequently located in the broncho-vascular bundles, interlobular septa, and alveoli, and there is frequent vascular involvement in the form of granulomatous angiitis. While most granulomas may spontaneously or with treatment disappear, some along the broncho-vascular bundles and interlobular septa often become fibrotic. In the chronic fibrotic stage, the upper lobes of the lungs become scarred and contracted, with fibrous bands seen between the hilar regions and pleura. Cystic changes and, occasionally, honeycombing are seen in the lung. Granulomatous vascular involvement is frequently seen in the lung. The microangiopathy of sarcoidosis is characterized by endothelial injury and multilamellation of the basement membrane of microvessels and has been observed electron microscopically in the heart, lung, eye, and skin specimens. The pathogenetic correlation between granulomas and microangiopathy remains to be resolved.

## 1 Introduction

Sarcoidosis is a multisystem disease that affects multiple organs and is characterized by non-caseating epithelioid cell granulomas in the affected organs (1). The precise etiology of sarcoidosis remains unknown, and many pathogens, including mycobacteria, have been implicated over the years.

In Japan, *Cutibacterium acnes* (formerly known as *Propionibacterium acnes*) is the only bacterium that has consistently been isolated from sarcoid lesions (2), and its presence has been confirmed by molecular studies, including PCR and *in situ* hybridization (3–5). Negi et al. (6) demonstrated the localization of *C. acnes* in granulomas, and Eishi (7) subsequently reviewed the etiologic link. However, outside of Japan, other microorganisms, including mycobacterial antigens, have also been implicated, and the etiology of sarcoidosis remained unresolved until date (8–11). Although the etiology of sarcoidosis remains unresolved until now, Sawahata et al. (12) recently reviewed latent microbial reactivation and immune dysregulation in sarcoidosis, focusing on *C. acnes*.

Sarcoidosis can affect multiple organs, including the lungs, lymph nodes, heart, eyes, and skin. Pulmonary involvement is of major clinical significance, as the lesions could progress to intractable fibrosis and cyst formation in the lungs. This review describes the pathological diversity of sarcoid lesions in the lungs, with particular emphasis on pulmonary fibrosis and vascular involvement, mainly based on autopsy of cases of Japanese patients with sarcoidosis.

## 2 Incidence and characterization of sarcoidosis in Japan

Ethnic differences are known to exist in the clinical features of sarcoidosis. Recent epidemiological surveys in Japan (13) have revealed that sarcoidosis occurs at an incidence of 1.01 per 100,000 population in Japan with a male-to-female ratio of 0.73:1.28. Morimoto reported a peak prevalence of sarcoidosis between 20 and 34 years of age in men and between 50 and 69 years of age in women (13). The organs most frequently affected, in addition to the lungs, are the eyes, skin, and heart. Sawahata et al. (14, 15) analyzed the relationship between the stage of illness based on diagnostic imaging findings and age and found that stage 1 or 2 predominantly occur in persons 45 years old or under and that stage 3 and 4 diseases predominantly occur in the elderly age group.

Iwai et al. (16, 17) investigated the autopsy findings of sarcoidosis in Japan between 1958 and 1989 and reported that both the distribution of granulomas in the body and the fatality rate tended to be higher among cases with cardiac sarcoidosis. Tachibana conducted autopsy investigations of sarcoidosis in patients who had died at 80 years later and reported that a high percentage of the patients had died from cardiac lesions (77%), and that malignant neoplasm was the most frequent cause of death after sarcoidosis in these patients (18).

## 3 Biopsy techniques for the diagnosis of pulmonary sarcoidosis

A Case-Controlled Etiologic Study of Sarcoidosis conducted in 2005 showed that biopsies of different sites of the body had been performed to make a definitive diagnosis of sarcoidosis (19). Biopsies within the thoracic cavity are the most frequently performed to identify non-caseating epithelioid cell granulomas. Since granulomas associated with various diseases can be detected in various organs, a differential diagnosis of sarcoidosis from other granulomatous diseases is necessary (20). The scalene lymph node biopsy was often used as a diagnostic procedure in the past, and then, the biopsy of the mediastinal lymph nodes by endobronchial ultrasound-guided transbronchial needle aspiration (EBUS-TBNA) has been often applied (21). Although granulomas can be identified and clearly diagnosed by EBUS-TBNA, evaluation of the entire lymph node structure is often difficult. At present, transbronchial lung biopsy and transbronchial lung cryobiopsy are the most frequently used procedures to diagnose pulmonary sarcoidosis (22).

## 4 Morphological characteristics of the granulomas

**Figure 1:**
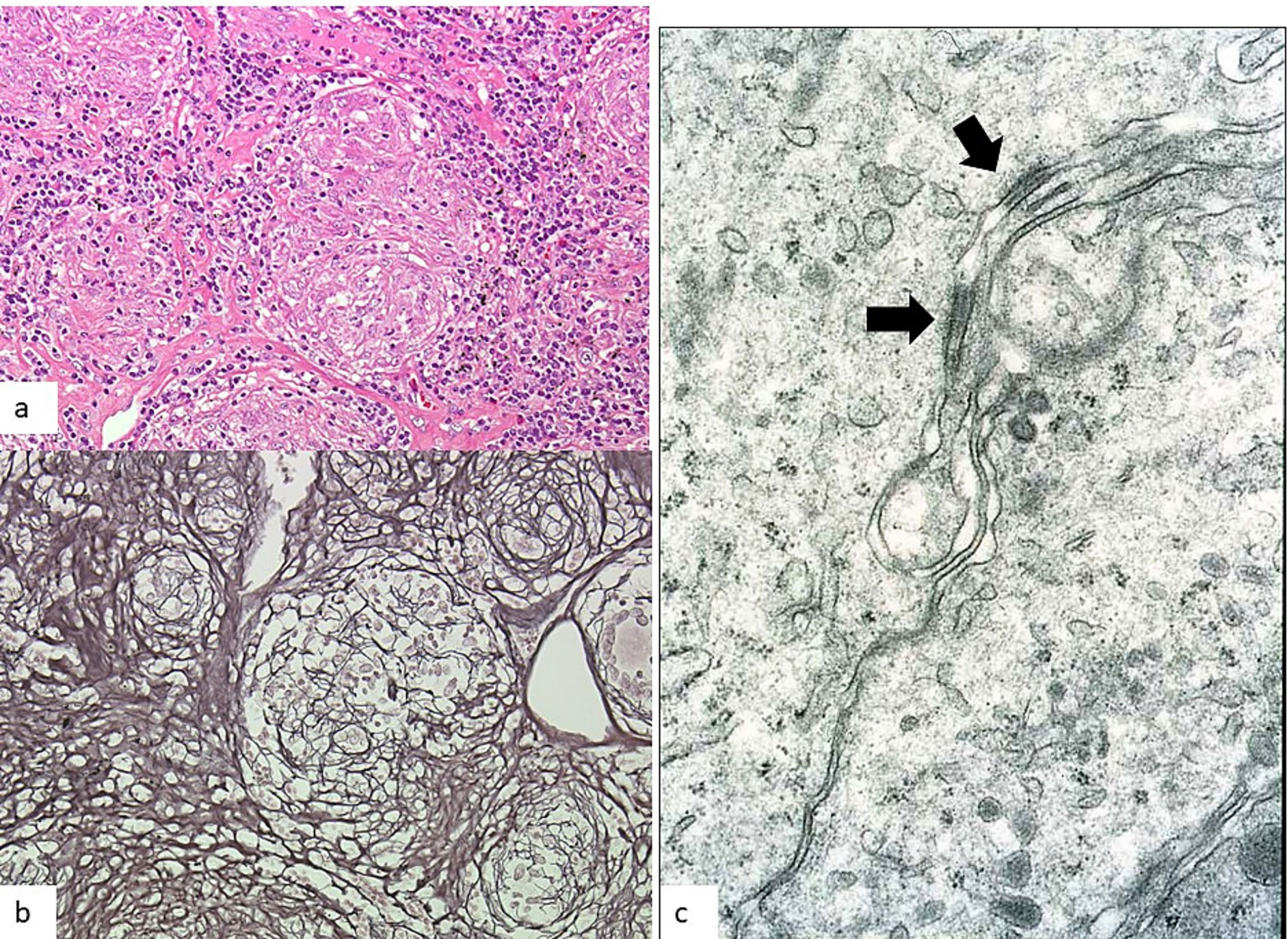
Epithelioid cell granuloma in the mediastinal lymph node in a case of sarcoidosis. **(a)** Epithelioid cell granuloma measuring 200 μm in diameter (hematoxylin–eosin stain, × 20). **(b)** Argyrophilic fibers encircling a granuloma in a basket-like form (silver impregnation stain, × 20). **(c)** Subplasmalemmal linear densities (arrows) are visible between adjacent epithelioid cells (transmission electron microscopy, × 10,000).

**Figure 2:**
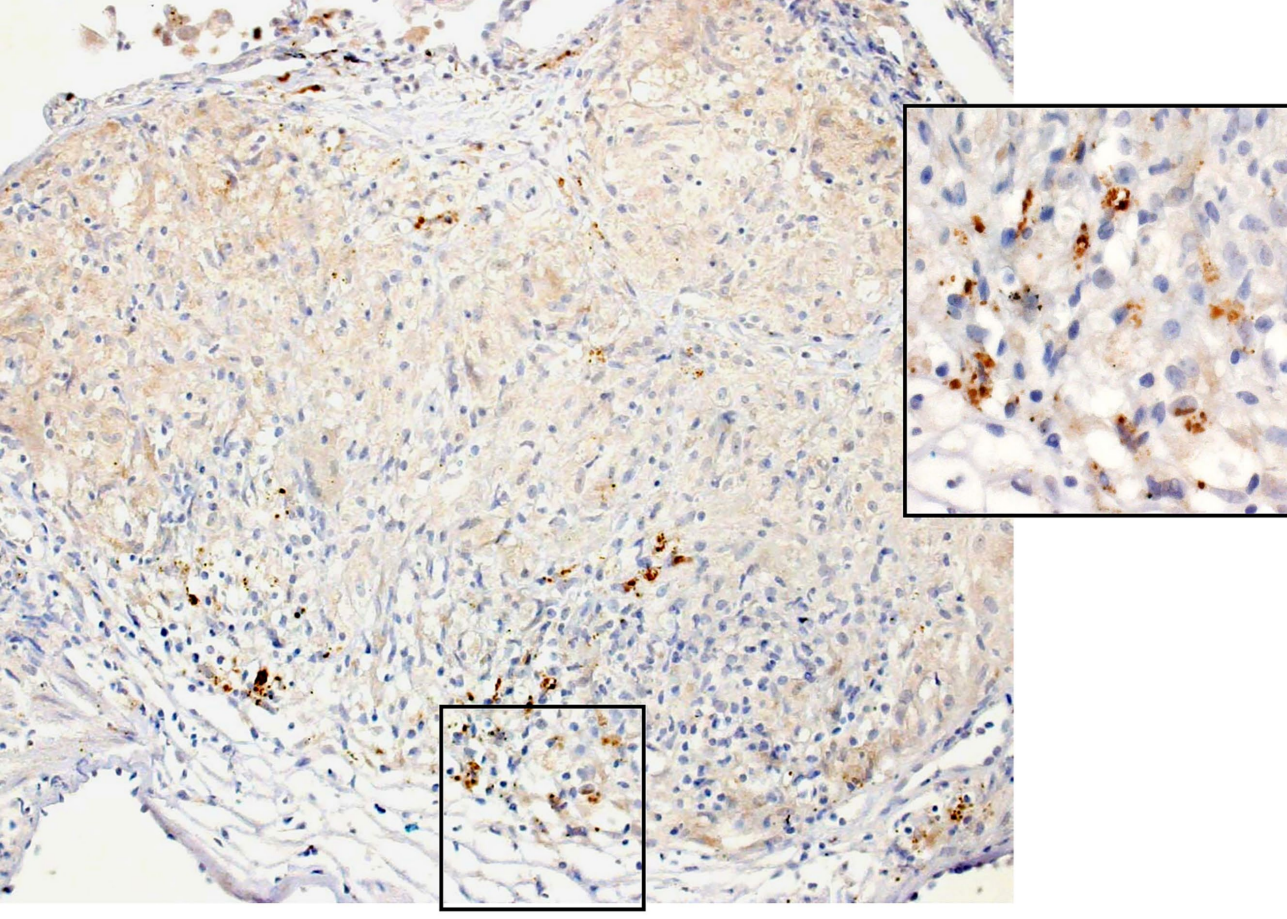
Non-caseating epithelioid cell granulomas in the lung that contain fine granular materials show positive immunohistochemical staining with a monoclonal antibody directed against lipoteicoic acid of *C. acnes* (immunohistochemistry × 20; Inset. × 40).

Granulomas in sarcoidosis are formed by the aggregation of macrophage-derived epithelioid cells as a result of a Th1 immune reaction induced by antigen presentation to T lymphocytes. This can be viewed as the morphological form of a host defense mechanism that isolates the body from the pathogens (11). Regulatory T cells and macrophages play a major role in granuloma formation (23, 24). Most of the granulomas in sarcoidosis consist of solitary granuloma measuring 200–300 μm in size or conglomerated ones composed of non-caseating epithelioid cells (25). Epithelioid cell granulomas are well demarcated from the surrounding tissues, and silver impregnation staining reveals argyrophilic fibers in a basket-like form. Well-developed subplasmalemmal linear densities are seen between adjacent epithelioid cells (Figure 1). The granulomas contain lymphocytes and Langhans giant cells in addition to epithelioid cells. CD4+ T lymphocytes are distributed within the granulomas, whereas CD8+ T lymphocytes are distributed at the margin of the granulomas. Granular substances have been detected in the sarcoid granulomas within the lung with the use of a monoclonal antibody to lipoteicoic acid of *C. acnes* (6, 7) (Figure 2).

### 4.1 Inclusion bodies in granulomas

**Figure 3:**
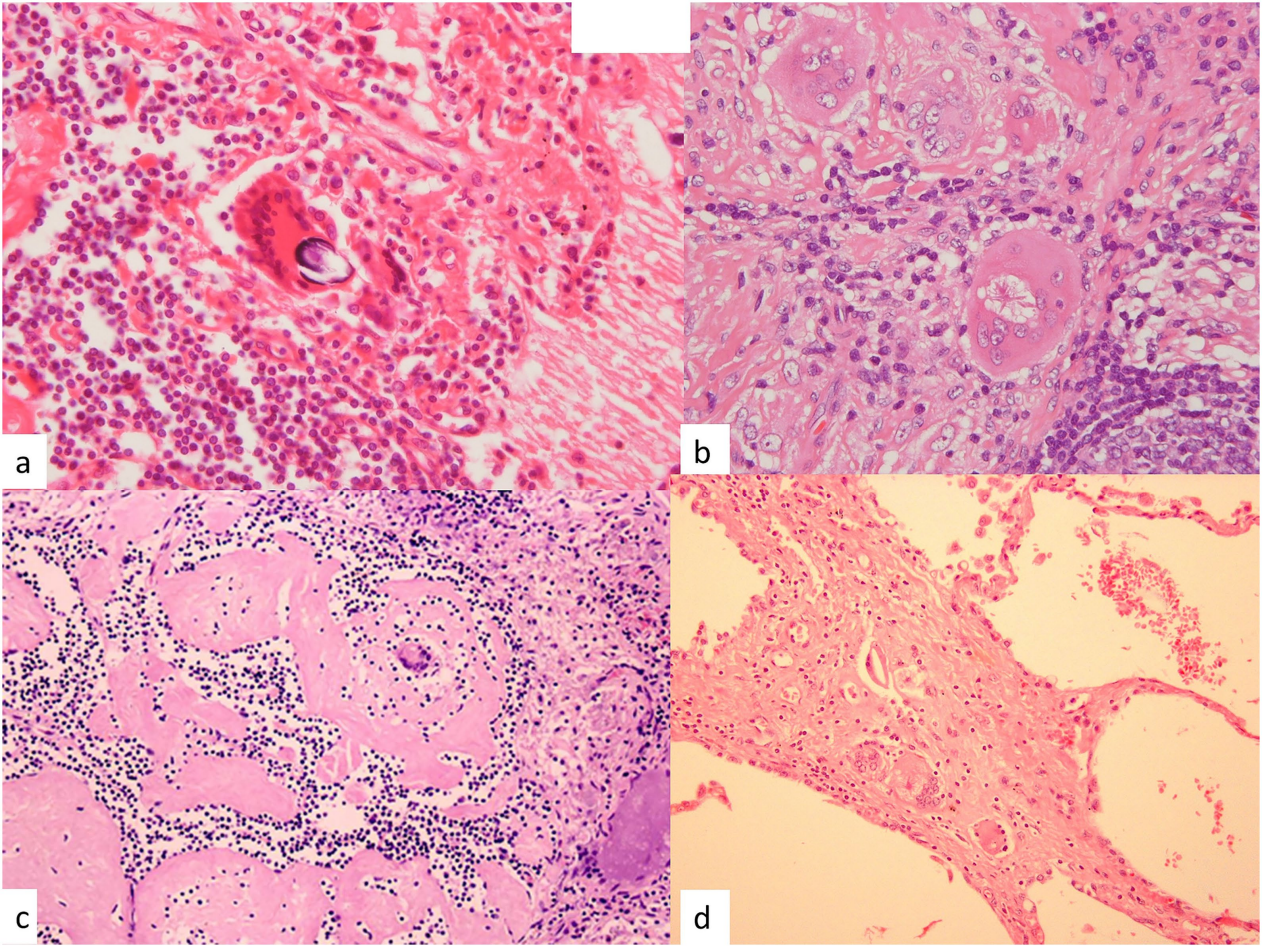
Variable morphologies of granulomas in the mediastinal lymph node and lung. **(a)** A giant cell with a Schaumann body in the mediastinal lymph node. (hematoxylin–eosin stain, × 40). **(b)** An asteroid body in a giant cell in an epithelioid cell granuloma. (Hematoxylin–eosin stain, × 40). **(c)** Hyalinization of granulomas with a multinucleated giant cell in the mediastinal lymph node. (Hematoxylin–eosin stain, × 10). **(d)** A fibrotic granuloma with a few giant cells in an alveolus. (Hematoxylin–eosin stain, × 20).

The giant cells in sarcoid granulomas often contain inclusion bodies such as Schaumann bodies (conchoidal bodies) that measure 25–200 μm in size and possess a lamellar structure containing iron and calcium (Figure 3a). Schaumann bodies are very common in sarcoidosis, but they are also seen in the granulomas associated with tuberculosis, berylliosis, and occasionally, hypersensitivity pneumonitis. Asteroid bodies are star-shaped inclusion bodies noted within multinucleated giant cells that measure 5–30 μm in size and are arranged in a radiating manner; they are seen in 2–9% of all sarcoid granulomas (Figure 3b). Asteroid bodies are composed of ubiquitin and myelin sheath-like membranous material and can also be found in tuberculosis, histoplasmosis, lepromas, and foreign body granulomas (25).

### 4.2 Hyalinization and necrosis of granulomas

Most sarcoid granulomas regress spontaneously but persist in the form of hyalinized nodules (Figure 3c). The granulomas in sarcoidosis have generally been described as non-necrotic epithelioid cell granulomas, but necrosis within the granulomas of sarcoidosis has also been reported in approximately 3–30% of cases (25).

### 4.3 Fibrosis of granulomas

Most granulomas in sarcoidosis resolve when the antigen is eliminated. Some granulomas develop fibrosis, often with remaining giant cells and lymphocytes (Figure 3d). The process of fibrosis of granuloma in pulmonary sarcoidosis may be induced by a dysregulated immune response. Th1-mediated immune reaction results in activation of fibroblasts. Various fibrogenic cytokines, such as TGF-β, TNF-α, IL-1β, and IL-6, are derived from the constituent cells of granuloma, and they are considered to be fibrogenic factors (23, 26–29).

## 5 Distribution of sarcoid granulomas in the lung

**Figure 4:**
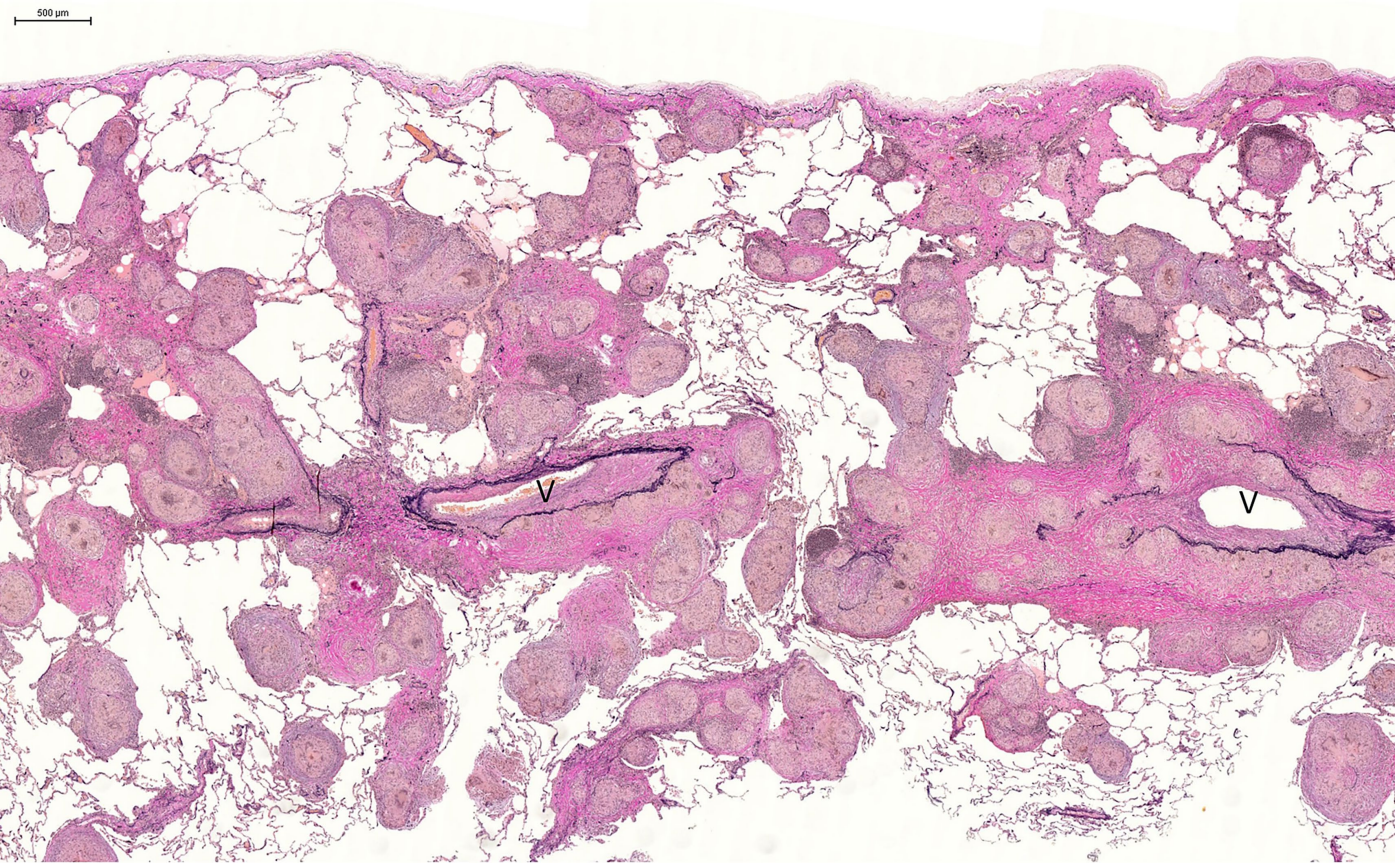
Characteristic distribution of granulomas in the lung, mainly in the interlobular septa, with vascular involvement (Elastica van Gieson stain, × 2.5). V, interlobular vein.

The granulomas in pulmonary sarcoidosis are primarily distributed along the lymphatic vessels, namely in the bronchovascular bundles, bronchiolar walls, interlobular septa, and the pleura (Figure 4). Granulomas are also detected within the lymphatic vessels and in the alveoli. Analyses of transbronchial lung biopsy specimens have revealed that the granulomas in the lungs are predominantly distributed in the upper lobes (30). In nodular sarcoidosis, granulomas involve the alveolar lumina and destroy the alveolar architecture, often leading to intense fibrosis. Moreover, the granulomas around the bronchovascular bundles sometimes compress the bronchial and bronchiolar tree and cause upper lobe collapse.

## 6 Fibrotic sequelae in pulmonary sarcoidosis

Akira et al. (31) conducted long-term follow-up computed tomography evaluation of pulmonary sarcoidosis and showed peribronchial conglomeration with shrinkage of the lung. We conducted a pathological investigation of the fibrosis pattern and remodeling of the lungs in 66 autopsy cases of sarcoidosis in Japan (32).

### 6.1 Histological patterns of fibrosis associated with granulomas in the lung

**Figure 5:**
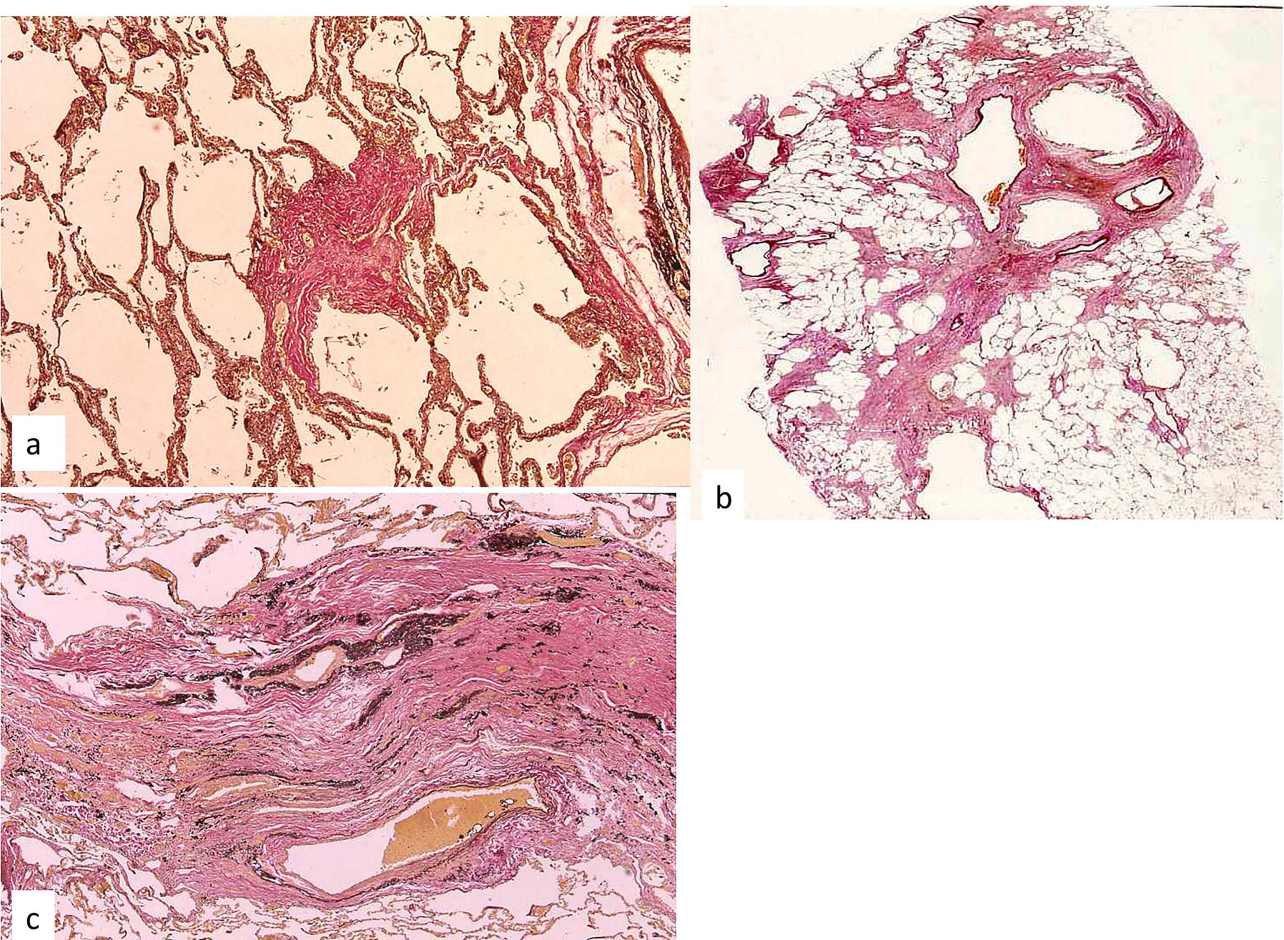
Fibrous sequelae of granulomas in the autopsied lung. **(a)** Stellate fibrosis at the respiratory bronchiole (Elastica van Gieson stain, × 5). **(b)** Bronchovascular fibrosis (Elastica van Gieson stain, × 1). **(c)** Collapse fibrosis along the bronchovascular bundle in the upper lobe (Elastica van Gieson stain, × 5).

The histological patterns of fibrosis associated with granuloma formations and remodeling of the lungs in sarcoidosis are shown in Table 1 (32). Stellate fibrosis is the most frequent type of fibrosis that occurs after the subsidence of a granuloma. Stellate fibrosis primarily occurs at the sites of granulomas along the bronchioles and pulmonary arteries and is also seen in the alveolar ducts (Figure 5a). Stellate fibrosis is the type of fibrosis seen in 95% of cases of pulmonary sarcoidosis and in 77% of cases in which the sarcoidosis does not predominantly affect the lungs, i.e., cardiac cases or nervous system. Bronchovascular fibrosis is observed primarily along the bronchioles and pulmonary arteries (Figure 5b) and was seen in 85% of cases of pulmonary sarcoidosis. Thick band-like collapse fibrosis (Figure 5c) was observed in 70% of cases of pulmonary sarcoidosis.

**Table 1:** Pathological features of the lung in 66 autopsy cases of sarcoidosis (32).

	Stellate fibrosis (%)	Band-like fibrosis (%)	Bronchovascular bundle fibrosis (%)	Collapse of bronchovascular bundle (%)	Upper lobe contraction (%)	Cavity (%)	Bulla (%)	Bronchiectasis (%)	Honeycombing (%)
Pulmonary sarcoidosis (*n* = 20)	19 (95)	17 (85)	16 (80)	14 (70)	13 (65)	9 (45) Aspergillus 8 (40)	11 (55)	13 (65)	10 (50)
Cardiac sarcoidosis (*n* = 31)	21 (68)	8 (26)	13 (42)	5 (16)	10 (32)	1 (3)	5 (16)	1 (3)	1 (3)
Neurosarcoidosis (*n* = 3)	3 (100)	0	3 (100)	2 (67)	2 (67)	0	0	0	1 (33)
Sarcoidosis without special organ involvement (*n* = 12)	8 (67)	3 (25)	6 (50)	4 (33)	1 (8)	0	0	1 (8)	1 (8)
Total 66 (female 45, male 21)	51 (77)	28 (42)	38 (58)	25 (38)	26 (39)	10 (15) Aspergillus 8 (12)	16 (24)	15 (23)	13 (20)

### 6.2 Bronchovascular bundle fibrosis and its sequelae

**Figure 6:**
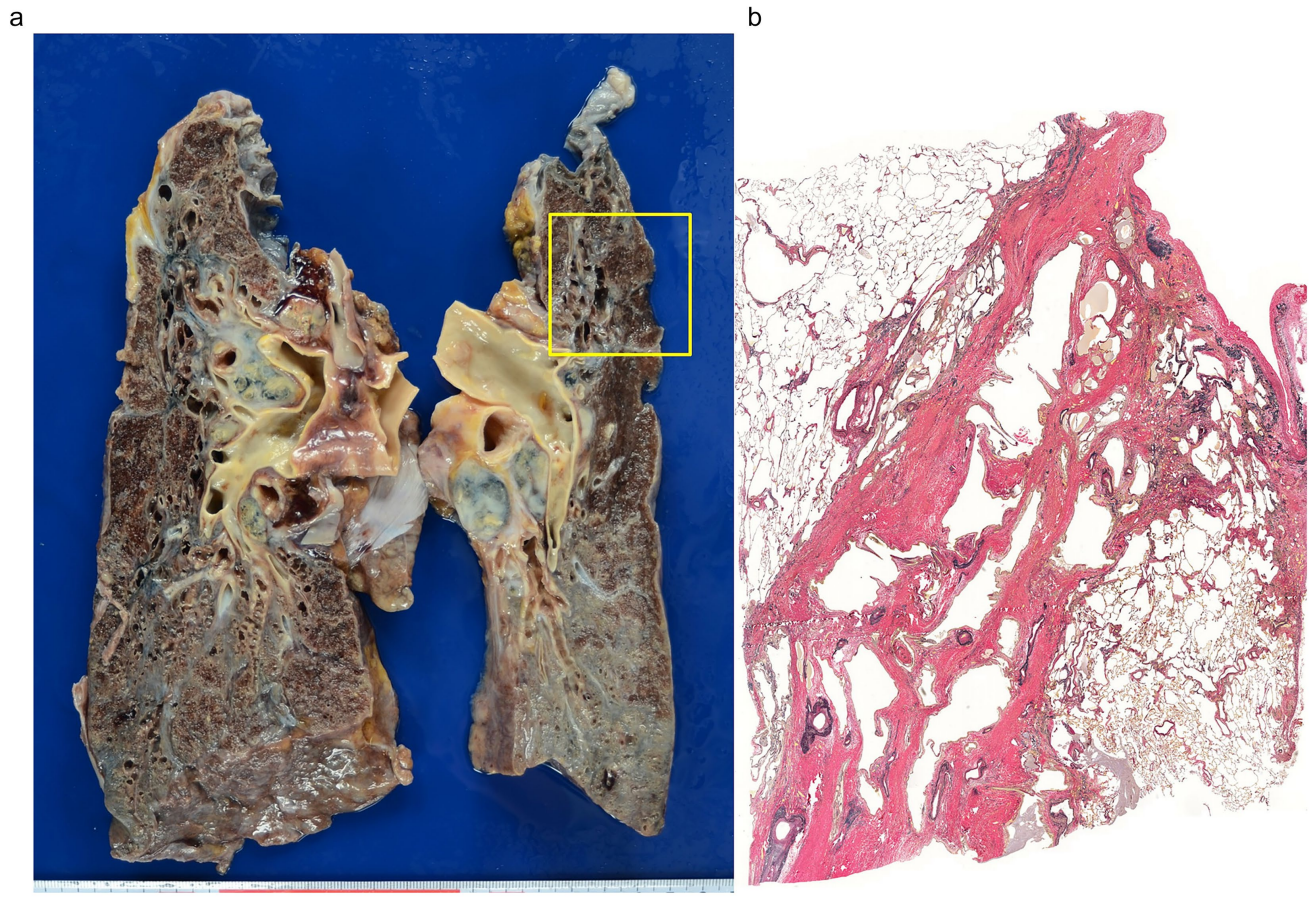
Upper lobe contraction in a 60-year-old man with a 40-year history of pulmonary sarcoidosis. **(a)** Marked bilateral shrinkage of the upper lobes with fibrous involvement of the hilar lymph nodes and bronchiolar dilatation. **(b)** Peribronchial dense fibrosis and dilatation of the bronchial lumina, extending from the hilar region to the pleura, indicated by the square in the left upper lobe (Elastica van Gieson, × 1).

Bronchovascular bundle fibrosis develops predominantly in the upper lobes, resulting in contraction of the upper lobes of the lungs. Sawahata et al. (33) recently reported characteristic central-peripheral fibrotic bands with upper lobe shrinkage in patients with long-standing sarcoidosis. Kawanobe et al. (34) also described central bronchial deformity on chest radiographs in pulmonary sarcoidosis as a crucial phenotype of upper lobe fibrotic changes. Upper lobe contraction has been estimated to occur in 65% of cases of pulmonary sarcoidosis and 32% of cases with cf. cardiac sarcoidosis. These cases present segmental bronchial stenosis secondary to hilar lymph node enlargement and bronchovascular bundle fibrosis (Figures 6a,b). Among the 13 cases of pulmonary sarcoidosis that presented with upper lobe contraction, 9 cases exhibited cavity formation as well. These nine cases were accompanied by *Aspergillus*. Cyst formation was noted in 11 cases (55%) of pulmonary sarcoidosis (Table 1). The cystic lesions are likely a result of check-valve mechanisms that develop as a result of advanced fibrosis of the wall of the proximal bronchi.

### 6.3 Honeycomb lesions in pulmonary sarcoidosis

**Figure 7:**
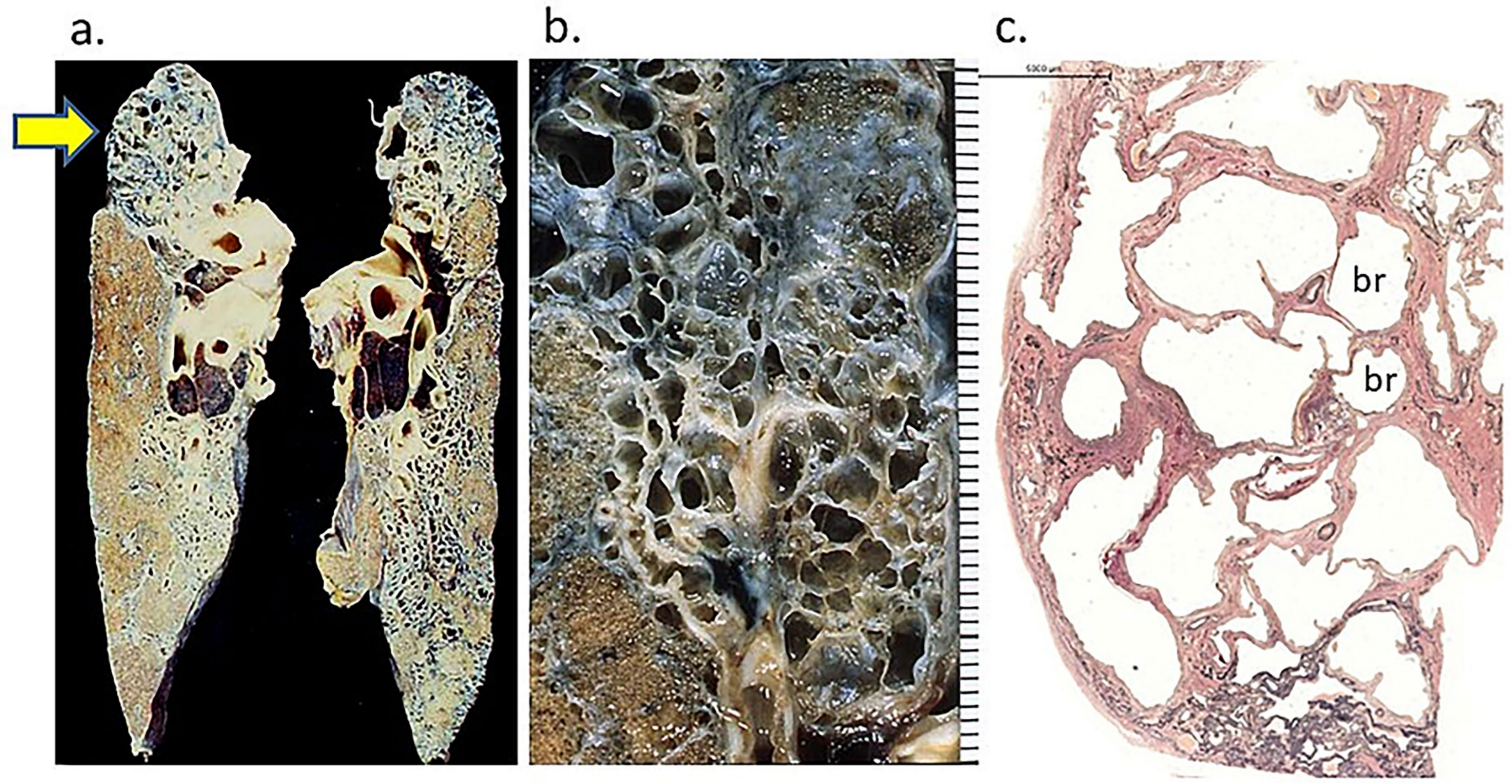
Bilateral upper lobe contraction with honeycomb-like lesion in a 40-year-old woman with a 27-year history of pulmonary sarcoidosis. **(a)** Honeycomb-like lesion involving almost the entire upper lobes bilaterally. **(b)** Close-up view of the right upper lobe showing a honeycomb-like appearance. **(c)** Pulmonary lobules containing irregularly sized cystic lesions, probably connected to the bronchioles but without perilobular alveolar collapse, different from the pattern of honeycombing observed in idiopathic pulmonary fibrosis (IPF)/usual interstitial pneumonia (UIP) (Elastica van Gieson stain, × 1.5).

The honeycomb lesions in sarcoidosis are not confined to the lower lobe of the lung and often predominate in the upper lobe (Figures 7a,b) (35). Histologically, the normal alveolar architecture is lost, replaced by enlarged airspaces, connecting to bronchioles without the obviously collapsed perilobular alveoli, seen in usual interstitial pneumonia (UIP) (Figure 7c). However, Shigemitsu et al. reported (36, 37) some cases of pulmonary sarcoidosis showing honeycombing with fibroblastic foci in the lower lobe, resembling those of idiopathic pulmonary fibrosis.

Coexistence of sarcoidosis and interstitial pneumonia has been reported (38, 39). Tachibana also reported acute exacerbation occurring in cases with lower lung-dominant sarcoidosis (40).

## 7 Vascular involvement in pulmonary sarcoidosis

### 7.1 Granulomatous angiitis and its sequelae

**Figure 8:**
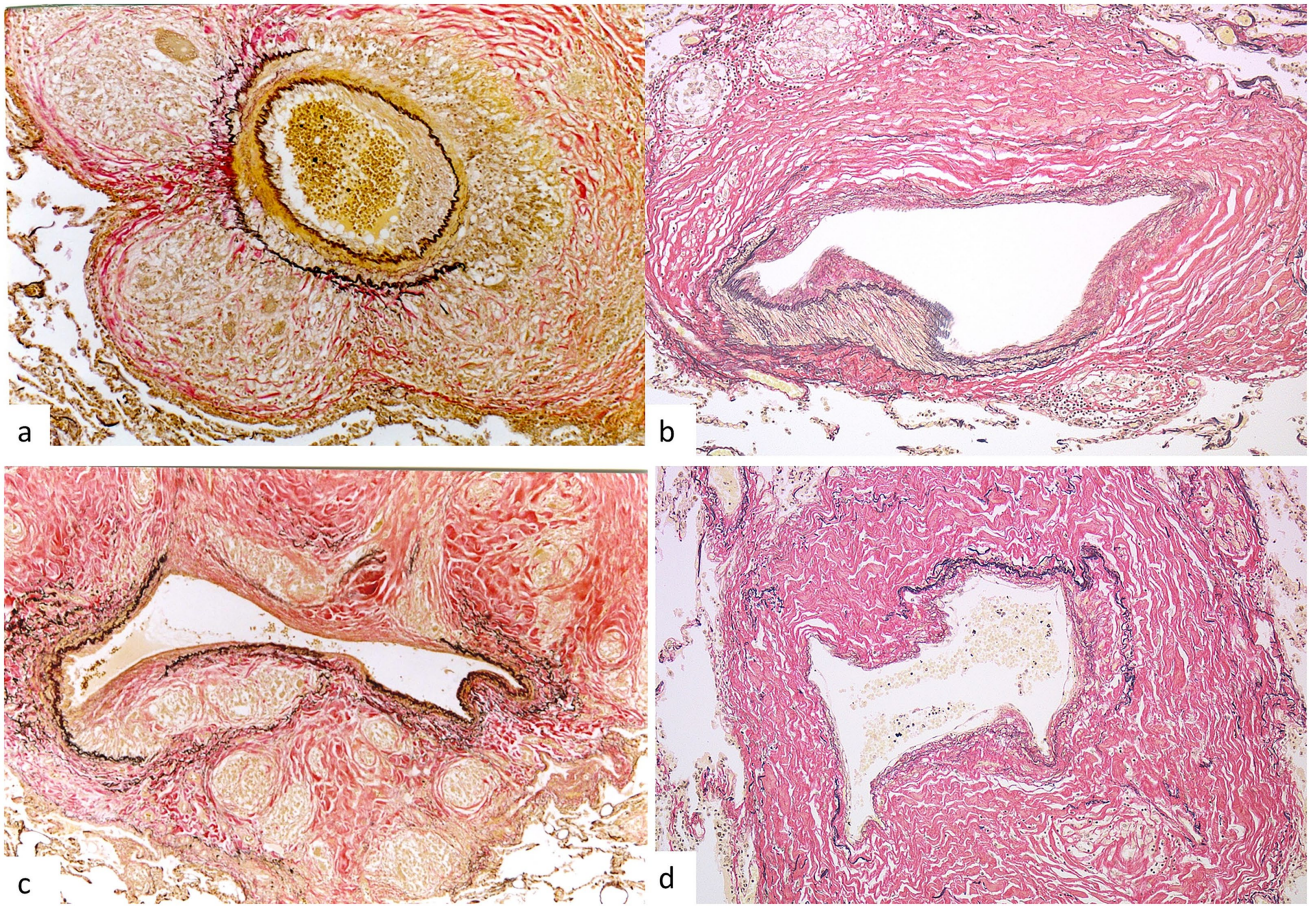
Granulomatous angiitis in pulmonary sarcoidosis. **(a)** Granulomatous involvement of a medium-sized pulmonary artery with destruction of the media and outer elastic lamina (Elastica van Gieson stain, × 10). **(b)** Scarred lesion resulting from granulomatous involvement of a pulmonary artery with dense deposition of collagen in the media and adventitia (Elastica van Gieson stain, × 10). **(c)** Granulomatous involvement of an interlobular vein with the destruction of the elastic lamina (Elastica van Gieson stain, × 10). **(d)** Scarring of an interlobular vein showing dense fibrosis in the venous wall and surrounding interstitium (Elastica van Gieson stain, × 10).

Granulomatous vascular involvement is one of the characteristic lesions of pulmonary sarcoidosis (41, 42). Granulomatous angiitis associated with sarcoidosis is characterized by granuloma formation in the vascular wall with destruction of the elastic fibers and/or smooth muscles of the vascular wall; no neutrophil infiltration or necrosis is observed. Vascular involvement with granuloma formation has been observed in transbronchial lung biopsy specimens (30), as well as autopsy specimens (42). Granulomatous involvement and its sequelae extend from the larger blood vessels, namely the pulmonary arteries and veins in the hilar region to the smaller blood vessels throughout the lung, with particularly marked involvement of the interlobular veins and intralobular arteries (42) (Figure 8). Scarring of the vascular wall resulting from healing of granulomatous angiitis was often noted in autopsied lungs, without the regeneration of the elastic fibers and smooth muscles of the vascular wall destroyed by granuloma formation and prominent deposition of collagen fibers.

Pulmonary hypertension in sarcoidosis has been known as sarcoidosis-associated pulmonary hypertension (SAPH) and is an important complication of advanced sarcoidosis (43). SAPH may be induced by multifaceted causes, such as granulomatous angiitis, veno-occlusive disease, pulmonary embolism, interstitial lung disease, and extrapulmonary disease, including left ventricular systolic and diastolic dysfunction. Thus, the cases with granulomatous vascular involvement must be carefully followed up to develop into pulmonary hypertension.

### 7.2 Microangiopathy in sarcoidosis

**Figure 9:**
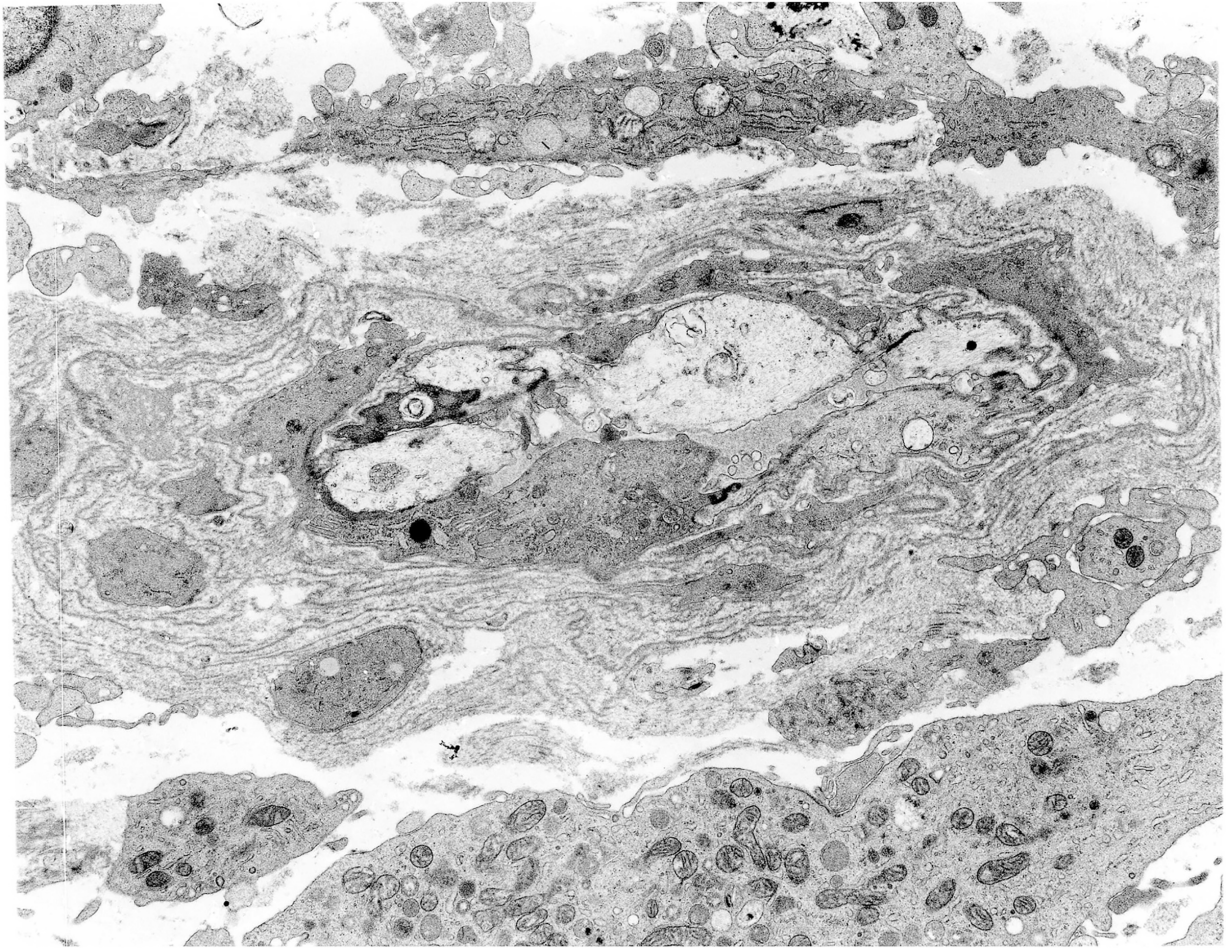
Microangiopathy of a capillary in the bronchial mucosa. Swelling of endothelial cells with multilayering of the basement membrane of a capillary in the bronchial mucosa (×10,000).

Microangiopathy in sarcoidosis has been observed clinically in the form of vascular lesions in the ocular fundus, a hexagonal pattern of dilatation of the bronchial mucosal vessels, and Raynaud’s phenomenon in the skin. Electron microscopic observations have revealed endothelial cell degeneration and multilayering of the basement membranes of small blood vessels (30, 44, 45) (Figure 9). Microangiopathic changes have been observed in the myocardium, skin, skeletal muscles, and bronchial mucosa in patients with sarcoidosis. In regard to the etiology of microangiopathy associated with sarcoidosis, it has been considered that vascular endothelial cell growth factors released from the constituent cells of granuloma and their receptors in the microcirculation system could be involved (46). However, further studies of microangiopathy are needed.

## 8 Conclusion

**Figure 10:**
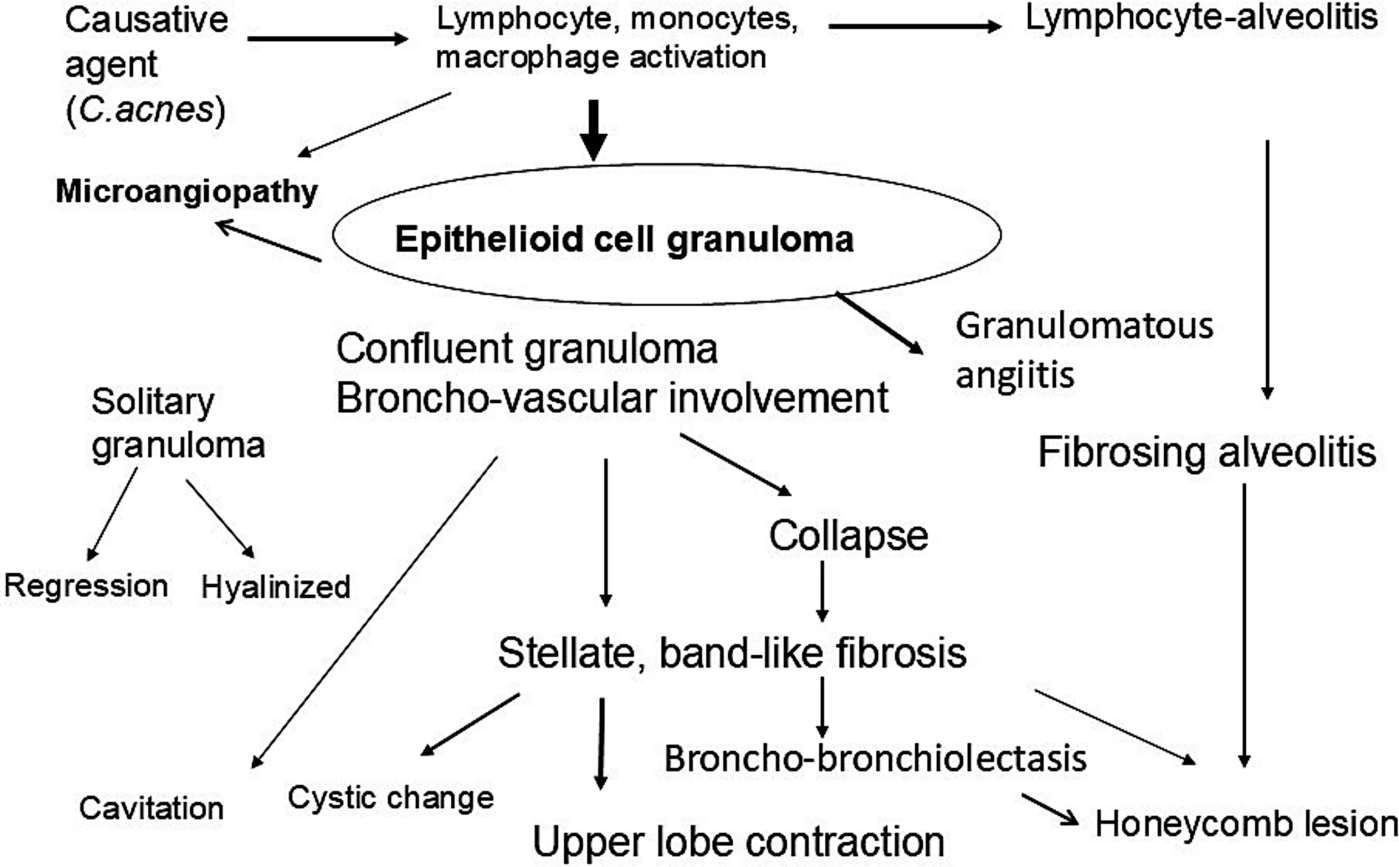
Sequelae of granuloma formation in pulmonary sarcoidosis. Some granulomas regress, while confluent granulomas involving the broncho-vascular bundles often progress to stellate or band-like fibrosis and collapse, resulting in bronchiectasis, upper lobe contraction, honeycombing, cyst formation, and cavitation.

I acknowledge that this pathological review of pulmonary sarcoidosis is limited to the Japanese patients, especially regarding the Japanese autopsy cases of sarcoidosis. In this paper, we have described the pathological features in the lung in patients with sarcoidosis, focusing on various fibrotic sequelae of epithelioid cell granulomas and remodeling of the lung. Granuloma-derived fibrosis predominantly involves the upper lobe bronchi from the hilar region to the pleura, resulting in upper lobe contraction and occasionally, formation of honeycomb-like lesions. Figure 10 shows a scheme of multifaceted pulmonary lesions derived from granulomas in sarcoidosis. Further research is necessary to resolve the process from granuloma to fibrosis and to prevent progressive fibrosis in pulmonary sarcoidosis.
